# Immune-driven remodeling in calcific aortic valve disease: mechanisms and immunotherapeutic opportunities

**DOI:** 10.3389/fimmu.2026.1906690

**Published:** 2026-10-19

**Authors:** Xi Zhang, Yi Si, Ling Liao, Yuxiang Wu

**Affiliations:** Institute of Intelligent Sport and Proactive Health, Department of Health and Physical Education, Jianghan University, Wuhan, China

**Keywords:** calcific aortic valve disease, calcification, immune cell, mechanisms, therapy, valvular interstitial cells

## Abstract

Calcific aortic valve disease (CAVD) is a prevalent degenerative valvular disorder in the aging population, for which no pharmacological therapy has proven effective in halting or reversing disease progression. Over the past decade, CAVD has been increasingly recognized not as a passive degenerative process of calcium deposition, but as an active, inflammation-driven disease characterized by osteogenic remodeling. This process is initiated and sustained by endothelial injury, lipid retention, immune cell infiltration, and phenotypic transition of valvular interstitial cells. Growing evidence indicates that innate and adaptive immune responses may contribute to disease amplification. Innate immune cells propagate local inflammatory signaling through inflammasome activation, extracellular vesicle communication, protease release, and neutrophil extracellular trap formation, while adaptive immune cells further shape disease progression by sustaining cytokine networks that promote fibrosis and osteogenic reprogramming within the valvular microenvironment. Based on these mechanistic insights, this review further discusses emerging translational strategies aimed at targeting key pathogenic processes in CAVD, including modulation of inflammatory signaling, restoration of adaptive immune homeostasis, regulation of extracellular matrix remodeling and antifibrotic pathways, inhibition of VIC osteogenic differentiation, as well as development of patient stratification and local drug delivery approaches. Collectively, these strategies may provide a conceptual framework for earlier, non-surgical interventions for CAVD.

## Graphical Abstract

**Figure:**
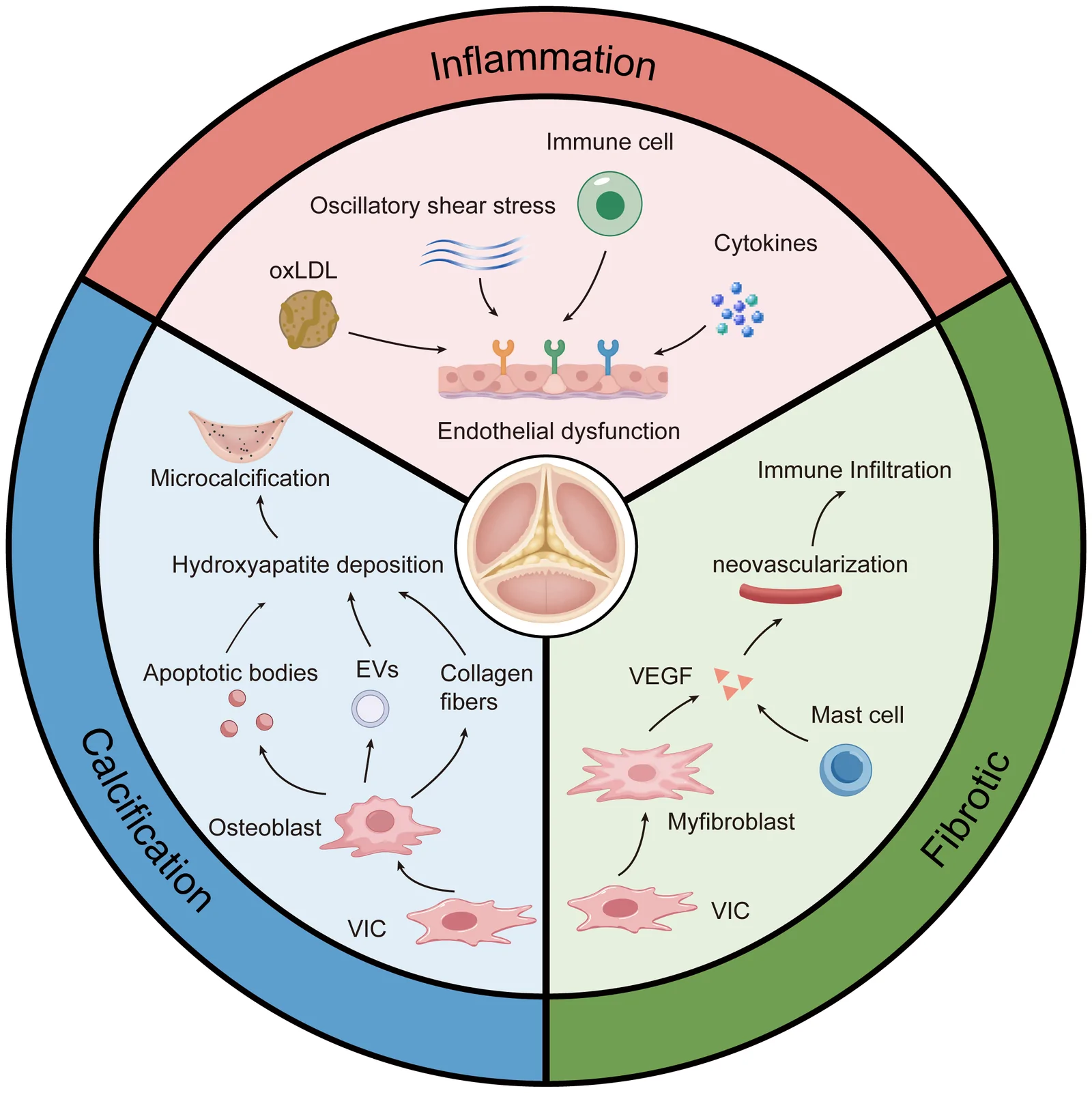
Pathological progression in calcific aortic valve disease. The illustration summarizes the progression of CAVD within the valvular microenvironment. Oscillatory shear stress, retained oxidized low-density lipoprotein, infiltrating immune cells, and proinflammatory cytokines converge on valvular endothelial cells, leading to endothelial dysfunction and establishment of a proinflammatory valvular microenvironment. During Fibrotic stage, valvular interstitial cells acquire an activated myofibroblast phenotype and secrete vascular endothelial growth factor, which drives neovascularization and sustains immune-cell infiltration, including mast cell accumulation, thereby reinforcing fibrotic remodeling. For calcification, valvular interstitial cells undergo osteogenic reprogramming into osteoblast-like cells, which release apoptotic bodies and extracellular vesicles and remodel collagen fibers, providing nucleation scaffolds for hydroxyapatite deposition and microcalcification that ultimately progress to established calcific nodules. CAVD, calcific aortic valve disease; EVs, extracellular vesicles; oxLDL, oxidized low-density lipoprotein; VEGF, vascular endothelial growth factor; VIC, valvular interstitial cell.

## 1 Introduction

Calcific aortic valve disease (CAVD) is a major age-related valvular disorder and an increasing challenge for cardiovascular and geriatric medicine (1–3). Its prevalence rises markedly with advancing age, and clinically significant stenotic disease affects a substantial proportion of older adults (1, 4). Pathologically, CAVD is characterized by progressive leaflet thickening, extracellular matrix (ECM) remodeling, fibrosis, and mineralization (4–7). As leaflet stiffness and valve obstruction worsen, patients are at risk of heart failure, syncope, and increased mortality (3, 4). Given ongoing population aging, the clinical and socioeconomic burden imposed by CAVD is expected to rise further (1, 2, 8).

Once largely regarded as a passive consequence of aging, CAVD is now recognized as an active, cell-mediated disease involving mechanical stress, endothelial dysfunction, lipid retention, chronic inflammation, ECM remodeling, and multicellular crosstalk (5–7). Within this pathological framework, activation and osteogenic reprogramming of valvular interstitial cells (VICs) are considered major cellular processes associated with progressive valvular fibrosis and mineralization (9–11). Injury or dysfunction of valvular endothelial cells (VECs), lipid oxidation, and matrix degradation are associated with the recruitment and activation of monocytes, T cells, and other immune populations within diseased valves (12–16). These immune cells can influence VIC phenotype and the valvular microenvironment through cytokines, extracellular vesicles, proteases, and other mediators, although the strength of evidence for specific immune cell–VIC interactions varies substantially among cell types and experimental settings (17–21).

Despite substantial advances in understanding the pathobiology of CAVD, no pharmacological intervention has yet been validated to reliably delay or reverse disease progression (3, 4, 9–11). Definitive management of severe aortic stenosis (AS) therefore remains dependent on surgical or transcatheter valve replacement (3, 4). These therapeutic limitations have increased interest in defining the immune and stromal mechanisms that operate during potentially modifiable stages of CAVD and in identifying interventions that may act before advanced fibrocalcific remodeling becomes established (21–24).

Previous reviews have summarized inflammatory pathways or individual immune-cell populations implicated in CAVD. However, important questions remain regarding how immune-cell heterogeneity interacts with the changing valvular microenvironment across disease stages and how these interactions influence the timing and feasibility of therapeutic intervention. We further discuss sex-related differences, limitations of experimental models, therapeutic timing, patient stratification, and emerging strategies for targeted intervention. This framework is intended to distinguish established mechanisms from context-dependent or putative pathways and to clarify which immune-related targets currently have sufficient evidence for further translational investigation.

## 2 Historical perspective on immune cell involvement in CAVD

The recognition of immune involvement in CAVD marks a pivotal conceptual shift within this field. Histological and immunohistochemical analyses of valves previously categorized as degenerative AS first demonstrated lipid accumulation and immune cell infiltration within early-stage lesions (12). Activated T cells and interleukin-2 (IL-2) receptor expression were then identified in nonrheumatic stenotic valves (13), while oxidized low-density lipoprotein was detected within stenotic valve tissue (14). Together, these observations challenged the long-held view that valvular calcification is a terminal passive process and instead provided early evidence that immune activation and lipid oxidation accompany early CAVD lesions (12–14, 25, 26).

Subsequent investigations reinforced the association between inflammation and ectopic osteogenic remodeling. Osteopontin was detected in calcified human aortic valve lesions, where its levels are significantly associated with the extent of calcification and macrophage accumulation (25). The identification of heterotopic bone formation in calcified and ossified valves, coupled with lymphocytes, monocytes, mast cells and bone morphogenetic proteins, further supported that CAVD involves active tissue remodeling rather than passive calcium deposition alone (26, 27).

In the early 21st century, lipid retention was considered a plausible initiating trigger for CAVD, fueling expectations that lipid-lowering interventions could attenuate AS progression. However, the Scottish Aortic Stenosis and Lipid Lowering Trial, Impact on Regression (SALTIRE), did not show meaningful benefit from intensive atorvastatin therapy (28); the Simvastatin and Ezetimibe in Aortic Stenosis (SEAS) trial was neutral for simvastatin plus ezetimibe (29), and the Aortic Stenosis Progression Observation: Measuring Effects of Rosuvastatin (ASTRONOMER) trial did not confirm rosuvastatin-mediated slowing of stenosis progression (30). These neutral outcomes challenged the expectation that lipid lowering alone would substantially modify established AS and contributed to a broader shift toward investigating inflammatory signaling, VIC biology, ECM remodeling, and local microenvironmental regulation (9–11, 31, 32).

Recent work has moved beyond merely documenting inflammatory infiltration to dissecting functional crosstalk between immune cells, VECs, and VICs. Experimental and transcriptomic studies have implicated inflammatory cytokines, extracellular vesicles, proteases, and innate immune receptors in VIC activation and osteogenic remodeling. These findings form the framework for the current emphasis on immune–stromal crosstalk, which is discussed in greater mechanistic detail in Section 5-6. This stepwise evolution of the immune-inflammatory paradigm is summarized in **Figure 1**.

**Figure 1:**
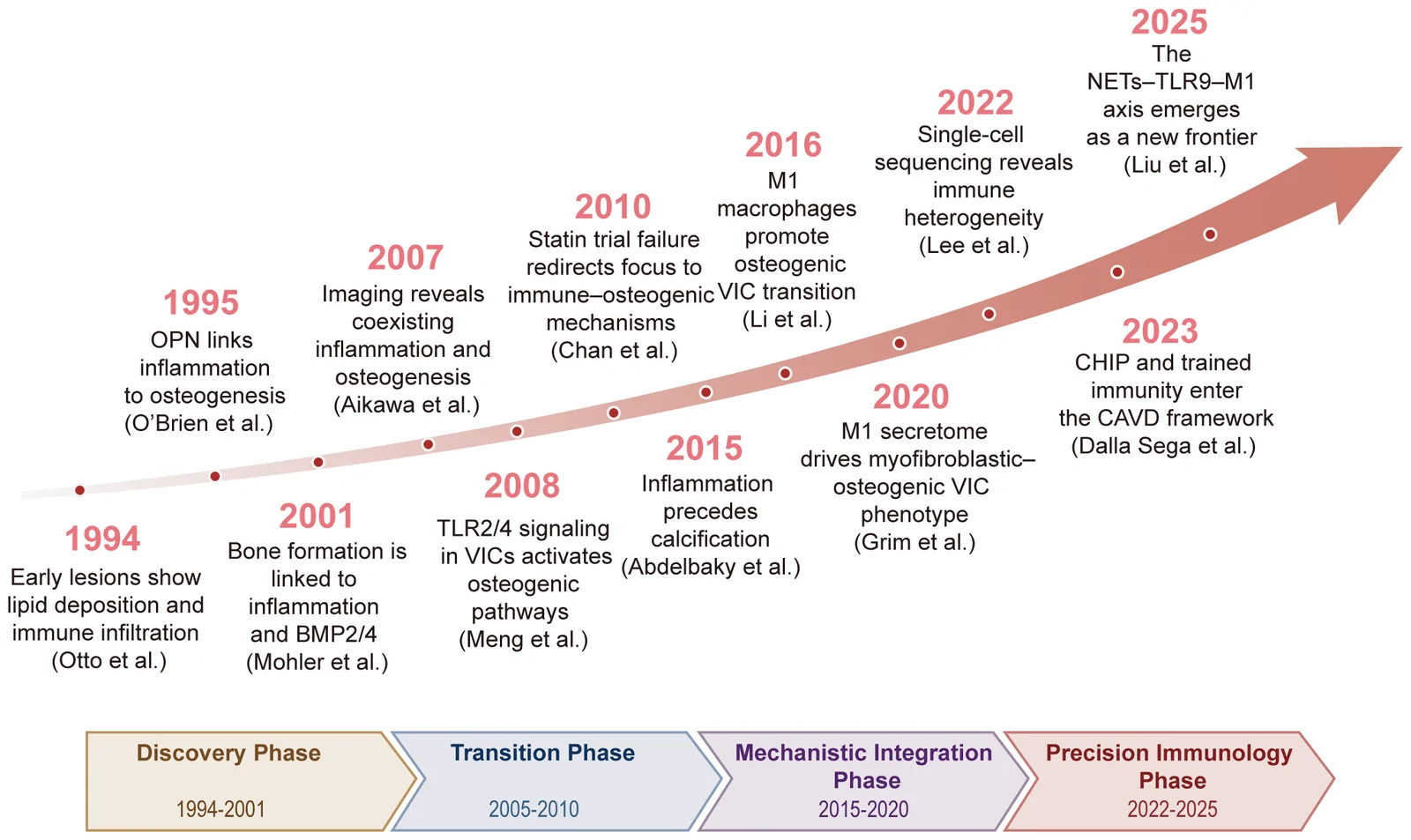
Milestones in research on immune-cell involvement in calcific aortic valve disease. The timeline summarizes the progression from early histological evidence of lipid deposition, macrophage infiltration, and T-cell infiltration to the recognition of inflammatory–osteogenic disease mechanisms, single-cell analysis, inflammasome biology, trained immunity, and NET-related mechanisms. The lower phase bar divides this progression into the discovery phase, transition phase, mechanistic integration phase, and precision immunology phase. BMP2/4, bone morphogenetic protein 2/4; CAVD, calcific aortic valve disease; CHIP, clonal hematopoiesis of indeterminate potential; NETs, neutrophil extracellular traps; OPN, osteopontin; TLR, Toll-like receptor; VICs, valvular interstitial cells.

## 3 Progression of CAVD

CAVD involves interconnected pathological processes encompassing endothelial dysfunction, inflammation, fibrotic remodeling, osteogenic reprogramming, and mineralization (33–35). Although these processes are often presented as a sequential cascade from endothelial injury to fibrosis and calcification, available human and experimental evidence indicates substantial temporal and spatial overlap among them. Accordingly, the injury–inflammation–fibrosis–osteogenic remodeling–calcification sequence used in this review should be regarded as a conceptual framework rather than a strictly linear temporal progression. Disturbed flow, lipid retention, and oxidative stress are associated with VEC dysfunction, leukocyte adhesion, and local inflammatory activation (16, 36–39), while mechanical and biochemical changes within the valve microenvironment can influence VIC activation, ECM remodeling, and osteogenic phenotypes (40–44). Increased expression of osteogenic regulators, including RUNX2, BMP2, and alkaline phosphatase, is associated with mineralization and calcific nodule formation, although the relative contribution and temporal relationship of these processes may vary across disease stage and tissue context (40, 41, 45–47).

### 3.1 Endothelial inflammatory activation

Endothelial dysfunction and inflammatory activation are considered important early features of CAVD and may contribute to the establishment of a proinflammatory valvular microenvironment (16, 33, 34, 37, 39). VECs cover the valstress andet surface, sense hemodynamic shear stress, and sustain endothelial barrier homeostasis under physiological conditions (16, 34). Early CAVD lesions exhibit side-specific heterogeneity, with lipid deposition, inflammatory infiltrates, and nascent calcific nodules predominantly localized to the aortic fibrosa layer (12, 33). Pattern-recognition receptor signaling, particularly Toll-like receptor activation by retained lipids and damaged matrix components, provides one plausible mechanistic link between this lipid-rich microenvironment and innate immune activation within the valve (48). Single-cell transcriptomic analyses indicate that VECs in diseased valves exhibit inflammatory activation, apoptosis-related signatures, and endothelial-to-mesenchymal transition programs (33, 49). Aberrant shear stress and mechanosensitive signaling can disrupt nitric oxide-redox homeostasis in VECs (16, 34), while Piezo1-mediated mechanotransduction provides a potential link between matrix stiffness and pro-calcific VIC signaling (50).

Following endothelial dysfunction, VECs may acquire a more inflammatory phenotype and contribute to local regulation of the valvular microenvironment. Injured or activated VECs can upregulate vascular cell adhesion molecule 1 (VCAM-1), intercellular adhesion molecule 1 (ICAM-1), and E-selectin, thereby facilitating leukocyte adhesion and transmigration (36). Persistent mechanical strain, oxidative damage, and inflammatory signals have been associated with endothelial-to-mesenchymal transition, including reduced endothelial marker expression and increased mesenchymal marker expression (16, 33, 35, 49). These phenotypic changes may compromise endothelial barrier integrity and contribute to local inflammatory signaling and immune cell recruitment (16, 35).

Dysregulated lipid metabolism may further exacerbate endothelial dysfunction and local inflammatory signaling in CAVD. Increased endothelial permeability enables interstitial retention of low-density lipoprotein, lipoprotein(a), and oxidized phospholipids within valve tissues (37, 51–54). Smad2-dependent glycosaminoglycan elongation in valve interstitial cells can further enhance low-density lipoprotein binding to valvular proteoglycans, reinforcing interstitial lipid retention (55). These lipid products can promote monocyte recruitment, macrophage lipid accumulation, and sustain chronic inflammatory signaling (37, 51, 53, 54, 56). Injured VECs also communicate with immune cells and VICs via extracellular vesicles, while macrophage-derived extracellular-vesicle cargo has been reported to promote osteogenic responses in VICs (18, 42, 57, 58).

### 3.2 Interstitial activation and matrix remodeling

VIC activation and ECM remodeling are important components of the structural remodeling that accompanies CAVD progression. In healthy aortic valves, VICs maintain ECM homeostasis by regulating collagen fibers, elastic fibers, proteoglycans, and other matrix constituents (39, 59, 60). During CAVD, mechanical stress, inflammatory cytokines, and lipid-related signals can influence VIC myofibroblastic activation, ECM remodeling, and osteogenic phenotypic changes (16, 37–40).

Single-cell sequencing studies have uncovered substantial phenotypic heterogeneity among activated and transitional VIC subpopulations in diseased valves (32, 33, 38, 49). Human calcified valves contain inflammatory, myofibroblastic, and osteogenic VIC populations, while transitional VICs may express IL-6, sortilin, and lumican, consistent with overlapping inflammatory, matrix remodeling, and osteogenic programming (33, 38, 49, 61). In experimental VIC models, increased matrix stiffness and mechanosensitive pathways involving Piezo1 and YAP, together with Wnt/β-catenin signaling, have been associated with increased expression of RUNX2, BMP2, alkaline phosphatase, and other osteogenic mediators (46, 50, 62, 63).

ECM remodeling is increasingly recognized as an active component of CAVD progression rather than merely a passive consequence of tissue injury. Excessive collagen deposition and lysyl oxidase-dependent collagen crosslinking can increase valvular stiffness, which may further enhance VIC mechanotransduction and sustain fibrotic and osteogenic signaling (50, 62–65). Elastic fiber degradation, aberrant proteoglycan accumulation, and oxidized lipid retention contribute to a pathological microenvironment associated with persistent immune infiltration and inflammatory signaling (16, 37, 39, 65). Together, VIC activation and ECM remodeling provide an interface through which inflammatory, mechanical, and biochemical signals may converge on fibrocalcific remodeling (16, 37, 39–41).

Importantly, fibrotic remodeling and mineralization should not be considered strictly coupled or temporally inseparable processes. Myofibroblast-associated features may change during disease progression, and reduced expression of markers such as α-SMA or reduced matrix contractility does not necessarily indicate regression of established mineralization. Once mineral deposits and associated matrix alterations have developed, calcification may persist or progress despite changes in the myofibroblast phenotype (43, 65).

### 3.3 Osteogenic conversion and mineral deposition

Osteogenic reprogramming of VICs is an important component of the fibrocalcific remodeling associated with progressive CAVD and advanced aortic stenosis. Under sustained inflammatory, mechanical, oxidative, and lipid-related stress, activated VICs can acquire osteogenic or osteoblast-like features in experimental models (40, 41, 43, 66, 67). This phenotype is commonly characterized by increased expression of RUNX2, BMP2, alkaline phosphatase, osteocalcin, and osteopontin (25, 40, 41, 46). The osteoclast-regulatory protein osteoprotegerin has also been reported to inhibit aortic valve calcification and preserve valve function in hypercholesterolemic mice, pointing to a modulatory role for the receptor activator of nuclear factor-κB ligand (RANKL)–osteoprotegerin axis in valvular mineralization (68). Therefore, valvular mineralization in CAVD is increasingly understood as a biologically regulated process involving VIC phenotypic changes, extracellular matrix remodeling, and cell-derived nucleation mechanisms rather than passive calcium precipitation alone (9, 10, 39–41).

Mineral deposition can arise from microscale nucleation events within the diseased valvular microenvironment. Apoptotic bodies, extracellular vesicles, denuded collagen fibers, and damaged ECM provide scaffolds for calcium-phosphate crystal nucleation and hydroxyapatite deposition (42, 57, 69–71). Progressive expansion and coalescence of microcalcifications form macroscopic calcific nodules, leading to leaflet thickening, reduced tissue elasticity, and restricted mobility (43, 69). Established calcification can alter local mechanical conditions and may further reinforce VIC osteogenic signaling and maladaptive ECM remodeling (50, 62, 63).

Immune-inflammatory signaling remains persistently active throughout mineralization. Collectively, osteogenic remodeling and mineral deposition are prominent features of advanced fibrocalcific CAVD, although inflammatory, fibrotic, and mineralization-related processes can overlap substantially across disease stages (9, 10, 39–41). Once extensive calcification is established, isolated anti-inflammatory or antifibrotic interventions may have limited capacity to reverse established structural valvular damage (4, 9–11, 65). The conceptual framework linking endothelial injury, VIC activation, and osteogenic mineralization is illustrated in **Figure 2**.

**Figure 2:**
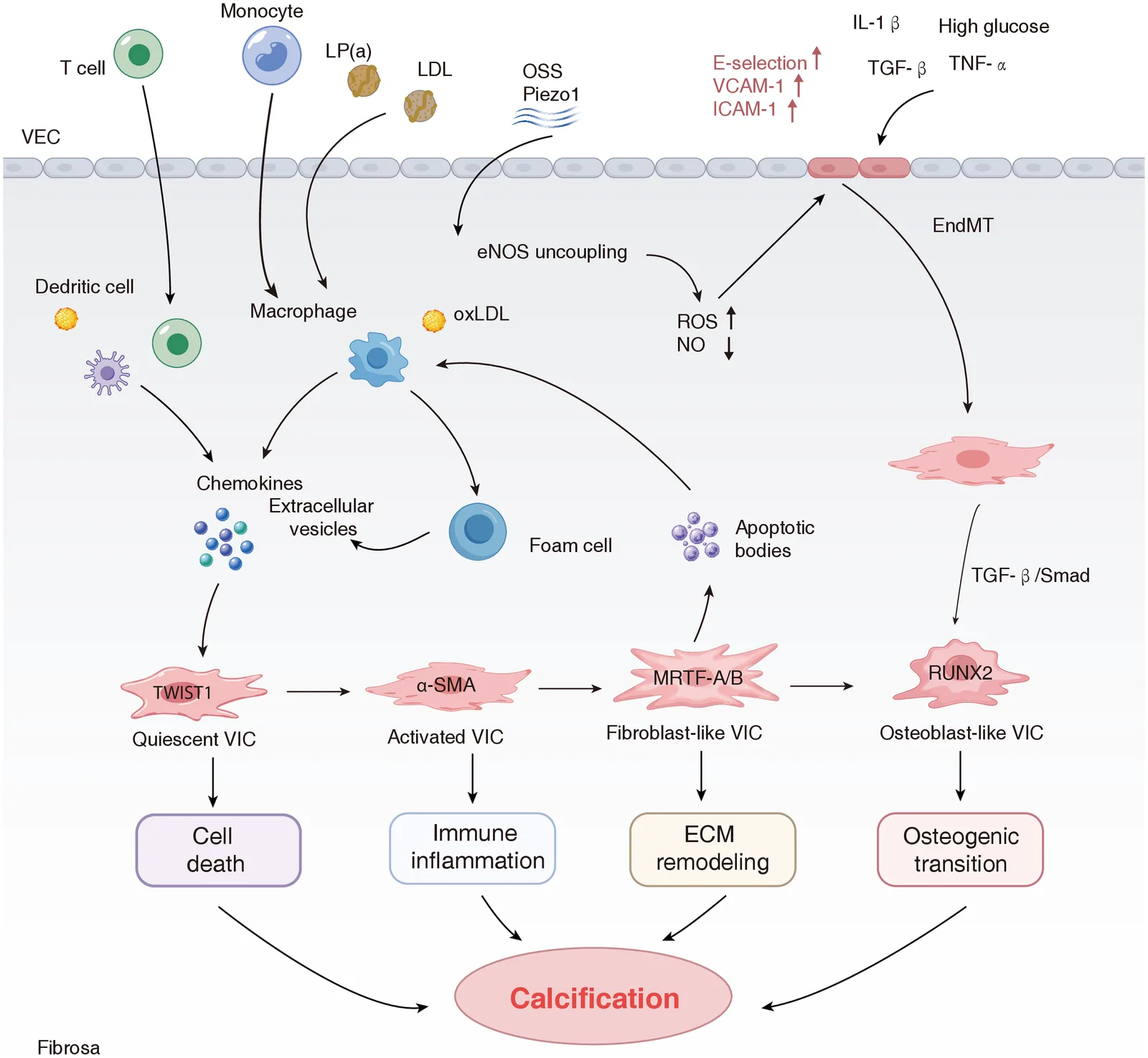
Endothelial injury, lipid retention, oxidative stress, immune-cell recruitment, EndMT, and extracellular matrix remodeling in calcific aortic valve disease. Disturbed shear stress, lipid retention, inflammatory cytokines (including IL-1β, TNF-α, and TGF-β), high glucose, and oxidative stress promote endothelial dysfunction, adhesion-molecule expression (E-selectin, VCAM-1, and ICAM-1), immune-cell recruitment, and EndMT. Piezo1-mediated mechanosensing and eNOS uncoupling with increased ROS and reduced NO further contribute to VEC dysfunction. The fibrosa, the aortic-side fibrous layer of the valve leaflet, is the region most prone to lipid retention, immune infiltration, and early calcification in CAVD. These signals cooperate with oxLDL, apoptotic bodies, extracellular vesicles, chemokines, foam-cell formation, and TGF-β/Smad signaling to drive VIC transition from quiescent TWIST1^+^ cells toward activated α-SMA+ and fibroblast-like MRTF-A/B^+^ states and then osteoblast-like RUNX2^+^ cells, ultimately promoting cell death, immune inflammation, ECM remodeling, osteogenic transition, and calcification. α-SMA, alpha-smooth muscle actin; CAVD, calcific aortic valve disease; ECM, extracellular matrix; eNOS, endothelial nitric oxide synthase; EndMT, endothelial-to-mesenchymal transition; E-selectin, endothelial selectin; ICAM-1, intercellular adhesion molecule 1; IL-1β, interleukin-1 beta; LDL, low-density lipoprotein; Lp(a), lipoprotein(a); MRTF-A/B, myocardin-related transcription factors A/B; NO, nitric oxide; OSS, oscillatory shear stress; oxLDL, oxidized low-density lipoprotein; Piezo1, piezo-type mechanosensitive ion channel component 1; ROS, reactive oxygen species; RUNX2, runt-related transcription factor 2; Smad, SMAD family protein; TGF-β, transforming growth factor-beta; TNF-α, tumor necrosis factor-alpha; TWIST1, twist family bHLH transcription factor 1; VCAM-1, vascular cell adhesion molecule 1; VEC, valvular endothelial cell; VIC, valvular interstitial cell.

## 4 Innate immune cells

VEC dysfunction, lipid retention, VIC activation, and aberrant ECM remodeling collectively create a local microenvironment that supports persistent innate immune activation (16, 33, 37–39). Innate immune cells contribute to CAVD through heterogeneous and partially overlapping mechanisms, with the strongest mechanistic evidence currently available for monocyte/macrophage-mediated inflammatory and immune-stromal interactions. Monocytes and macrophages represent the most extensively studied populations and have been implicated in multiple processes during CAVD (17, 22, 72–74). DCs provide a potential interface between innate sensing and adaptive immune activation, whereas neutrophils and mast cells may influence the valvular microenvironment through NET formation and protease-dependent tissue remodeling (19, 75–78). By contrast, evidence for NK-cell involvement remains largely transcriptomic or inferential (21, 79).

### 4.1 Monocytes and macrophages

Monocytes and macrophages are among the most extensively studied innate immune cell populations in CAVD and have been implicated in both inflammation and fibro-calcific remodeling (17, 22, 73). Under conditions of VEC dysfunction, lipid retention, and chemokine signaling, circulating monocytes can be recruited into the valvular interstitium and differentiate into heterogeneous macrophage states (73, 74). CAVD-specific studies implicate macrophages in inflammatory amplification, ECM remodeling, and pro-calcific VIC signaling, although the relative contributions of macrophage states to fibrosis and mineralization remain context-dependent (18, 72, 80–82).

Monocyte recruitment is mediated by chemokine signaling and is accompanied by production of TNF-α, IL-6, IL-1β, matrix metalloproteinases, and other inflammatory mediators (73, 83). These mediators can modulate inflammatory signaling in VICs, contribute to matrix remodeling, and reinforce immune–stromal crosstalk (80, 81). Cytokine alarmins may provide additional links between inflammation and VIC phenotypes: interleukin-33 (IL-33) induces differential phenotypic transitions in valvular interstitial cells and promotes the progression of nonrheumatic aortic valve stenosis (84). Patients with severe aortic stenosis exhibit elevated circulating intermediate monocytes, which correlate with systemic inflammatory burden and disease severity (85). Clonal hematopoiesis studies have further linked altered monocytic–macrophage inflammatory programming to severe aortic stenosis and outcomes after transcatheter aortic valve replacement (TAVR) (74, 86, 87). These clinical associations support the relevance of monocytic–macrophage inflammatory states to advanced AS but do not by themselves establish causality in valvular calcification.

Macrophage function in CAVD is highly phenotype- and context-dependent. Importantly, the conventional M1/M2 framework may not fully capture the spectrum of macrophage states present in diseased human valves. M1-like inflammatory macrophage states have been identified in lipid-rich and calcified valvular regions and are associated with proinflammatory and pro-calcific signaling (72). At the downstream signaling level, IL-6 signals through IL-6R–gp130–JAK2 to activate STAT3, TNF-α engages TNFR1–RIPK1/TRADD/TRAF2 to activate the TAK1–IKK–NF-κB axis, and IL-1β signals through IL-1R1/IL-1RAP via the MyD88–IRAK4–TRAF6 module to activate NF-κB. *In vitro* studies indicate that these pathways can promote osteoblast-like phenotypic changes in VICs with upregulation of TNF and IL6 transcription, thereby reinforcing paracrine inflammatory loops between macrophages and VICs (80, 81). M1 macrophage-derived extracellular vesicles can also deliver the tRNA-derived small RNA tsRNA-5006c, modulating VIC mitophagy and osteogenic differentiation (18). Metabolic reprogramming, increased lipid uptake, and foam-cell formation may further alter macrophage inflammatory and pro-calcific signaling (17, 22, 37, 88).

Beyond these biochemical cues, local mechanical conditions can also shape macrophage behavior in CAVD. Increased matrix stiffness and disturbed flow can modulate macrophage inflammatory polarization as well as phagocytic or pro-calcific functions through mechanosensitive pathways, although whether similar stiffness-dependent reprogramming occurs within stenotic valve microenvironments has not been directly examined (16, 50, 89).

Nevertheless, macrophage phenotypes in CAVD are unlikely to conform to a binary M1/M2 classification. Transcriptomic studies of human valves have identified multiple macrophage states associated with lipid handling, inflammatory signaling, tissue remodeling, and resolution-linked programs, suggesting that macrophage function may vary with disease stage and local microenvironment. IL-10 production and efferocytosis may contribute to inflammatory resolution, whereas chronic injury may favor macrophage programs associated with transforming growth factor-β (TGF-β) signaling and fibrosis. However, direct CAVD-specific causal evidence regarding the roles of distinct M2-like states in tissue repair or fibrosis remains limited (17, 22, 88, 90).

Overall, current evidence supports macrophages as important modulators of inflammation and fibro-calcific remodeling in CAVD, whereas the functions of specific macrophage states and their stage-dependent contributions remain incompletely defined.

### 4.2 Dendritic cells

DCs in CAVD may link local lipid oxidation, tissue injury, and danger-signal recognition to adaptive immune activation. They are sparse in healthy aortic valves but can be distributed in regions susceptible to disturbed shear stress, lipid retention, inflammatory infiltration, and calcified lesions, and their primary function may be antigen presentation and immune amplification rather than direct induction of VIC mineralization (23, 24, 75). Oxidized lipids, necrotic cellular debris, and degraded ECM components can promote DC maturation, MHC-II expression, costimulatory signaling, and interactions with T cells (24, 75). T cell aggregation and clonal expansion in stenotic valves are consistent with antigen-driven immune activation, although the relative contributions of antigen specificity and bystander activation remain incompletely defined (20, 91). SPP1 represents a potential molecular interface between immune activation and mineralization, as osteopontin has both matrix-regulatory and immunoregulatory functions (25, 92). Its expression correlates with DC, T cell, and macrophage infiltration as well as proinflammatory and pro-fibrotic VIC features (92). Mechanistically, SPP1 has been reported to engage integrin–FAK–PI3K/Akt signaling in VICs, a mechanosensitive axis that may link matrix remodeling to VIC survival and osteogenic competence, although direct evidence in human calcified valves remains limited (92).

Overall, current evidence mainly supports DCs as immune-activation bridges between tissue injury and adaptive immunity, whereas their direct contributions to VIC activation and valvular mineralization remain incompletely established.

### 4.3 NK cells

NK cells are innate lymphocytes with cytotoxic and immunoregulatory functions, yet their pathological roles in CAVD remain poorly defined. Single-cell and immune-infiltration analyses identify NK-like populations or signatures in diseased aortic valves, but these observations do not establish a causal role in calcification (32, 33, 38, 49, 79). Potential NK-cell effects on macrophages, T cells, VECs, and VICs through cytokine or cytotoxic programs remain biologically plausible, but direct CAVD-specific functional evidence is insufficient. In particular, transcriptomic abundance estimates should not be interpreted as proof that NK cells directly induce VIC osteogenic reprogramming (21, 33, 38, 49, 79). Functional interpretation is further complicated by phenotypic overlap between conventional NK cells and cytotoxic T-cell populations in transcriptomic and deconvolution datasets. Current evidence therefore supports describing NK cells as potential immune-network modulators rather than established drivers of valvular calcification (21, 79, 93). Valve-focused spatial and functional studies are required to define the distribution and specific roles of NK subsets in CAVD.

### 4.4 Mast cells

Healthy aortic valves contain few mast cells, whereas stenotic and calcified valves exhibit increased numbers of CD117^+^ mast cells within inflammatory infiltrates (76). Mast cell density correlates with stenosis severity, suggesting an association with established lesion progression rather than evidence of disease initiation (19, 76, 77). Mast cell effects may be partly mediated by degranulation-derived effector molecules. Activated mast cells release tryptase, chymase, cathepsin G, and angiogenic factors, which can contribute to ECM degradation, elastin fiber disruption, inflammatory amplification, and pro-fibrotic remodeling (19, 76). At the receptor level, mast cell-derived cathepsin G may contribute to valvular remodeling through elastin degradation and local angiotensin II generation, the latter engaging angiotensin II type 1 receptor (AT1R) on VICs to activate NF-κB–dependent inflammatory transcription (19). Mast cell-derived TGF-β can further engage TGF-β receptor (TβR)–ALK5 to activate Smad2/3–Smad4 signaling, promoting COL1A1 and ACTA2 expression and myofibroblastic VIC differentiation (19, 76, 77). Mast cells and myofibroblasts can also produce VEGF, potentially contributing to neovascularization and sustained immune-cell recruitment within diseased valves (77, 94, 95).

Overall, current evidence emphasizes mast cells as microenvironmental modifiers rather than primary osteogenic effectors in CAVD. Further mechanistic studies are needed to validate whether mast cell localization, degranulation activity, and protease function directly regulate fibro-calcific remodeling in CAVD.

### 4.5 Neutrophils

Neutrophils are rapid responders to tissue injury, yet direct evidence supporting their pathogenic role in CAVD remains more limited than for macrophages or T cells. Clinically, elevated neutrophil-to-lymphocyte ratio correlates with adverse outcomes after aortic valve replacement or TAVR, reflecting the prognostic value of systemic inflammation in valvular disease (96–99). However, this biomarker alone cannot confirm a direct causal role of neutrophils in valvular calcification (96). Neutrophil extracellular trap (NET) formation provides a potential mechanistic link between neutrophil activation and CAVD progression (100). In preclinical models, NETs can promote M1-like macrophage polarization through Toll-like receptor 9 (TLR9) signaling (78). This process is accompanied by upregulated TNF-α, IL-1β, BMP2, and RUNX2 (78). ECM composition also modulates neutrophil activity, with chondroitin sulfate-rich matrix restricting NETosis in three-dimensional CAVD models (101). Neutrophil elastase has also been implicated in valvular injury and pathological remodeling. In experimental models, exposure to neutrophil elastase can induce VIC inflammatory responses, apoptosis, and calcification (102). In apolipoprotein E (ApoE)-deficient mice, elastase inhibition mitigates valvular thickening and preserves cardiac function (102). Overall, current evidence supports potential roles for neutrophils in CAVD through NET–TLR9–macrophage signaling and elastase-associated VIC injury, although the relative importance of these pathways in human CAVD remains to be established (97, 98, 103–105).

## 5 Adaptive immune cells

Adaptive immune cell infiltration is a prominent feature of the immune microenvironment in diseased aortic valves. T cell infiltration, clonal expansion, and effector-memory phenotypes have been documented in calcified valves (20, 91, 106). B cells have also been detected in stenotic valve tissue, where their density was associated with disease severity (107). Adaptive immune cells may influence the valvular microenvironment through antigen presentation, cytokine secretion, antibody production, and reciprocal interactions with myeloid cells and VICs; however, the strength of causal evidence differs substantially among immune-cell subsets and signaling pathways (20, 21, 23, 24, 107). Comparative insights from atherosclerosis, where individual T cell subsets have been assigned distinct pathogenic and regulatory roles, may help contextualize the subset-level evidence emerging in CAVD (108).

### 5.1 CD4^+^ T cells

CD4^+^ T cells are among the earliest adaptive immune cells identified in CAVD. They are sparse in healthy aortic valves but accumulate within calcified lesions near lipid deposits, inflammatory infiltrates, and neovessels (20, 94). Under conditions of VEC dysfunction, oxidized lipid retention, and local antigen exposure, CD4^+^ T cell activation may be supported by antigen-presenting cells within diseased valves (24, 75). Activated CD4^+^ T cells may contribute to chronic valvular inflammation, matrix remodeling, and mineralization through cytokine-dependent immune–stromal crosstalk (20, 24, 109–111).

The Th1/IFN-γ signaling axis exemplifies the context-dependent nature of T cell–mediated effects in CAVD. In experimental models, increased Th1 cell abundance and IFN-γ/STAT1 signaling can accelerate disease progression, and systemic IFN-γ overexpression can exacerbate valvular calcification and dysfunction (109). Conversely, Th1-derived IFN-γ can inhibit macrophage NLRP3 activation and reduce the osteogenic effects of macrophage-conditioned medium on VICs, suggesting a potential negative-feedback mechanism (109). These seemingly opposing effects highlight the importance of cellular context and disease stage in interpreting IFN-γ signaling in CAVD.

The IL-17A signaling axis is another inflammatory pathway associated with CD4^+^ T cells and implicated in CAVD. IL-17A, interleukin-17 receptor A (IL-17RA), and RUNX2 expression are elevated in human calcified valves, and IL-17A/IL-17RA levels correlate positively with RUNX2 expression (110). Mechanistically, IL-17A can act on IL-17RA-expressing VECs to increase IL-6 and IL-1β production, thereby promoting local inflammatory signaling and immune-cell recruitment (110). Downstream of ligand binding, IL-17A engages IL-17RA and the adaptor ACT1–TRAF6 module to activate NF-κB–dependent inflammatory transcription, providing a receptor-proximal mechanism by which Th17-derived signals amplify local inflammation and fibrotic gene expression (110, 112). IL-17A is also associated with enhanced fibrotic gene expression and adverse matrix remodeling, whereas IL-17A neutralization can reduce valvular calcium deposition and RUNX2 expression *in vivo* (110). Beyond IL-17A, the Th17-associated cytokine IL-22 has been implicated as an additional pro-osteogenic mediator, promoting osteogenic differentiation of valvular interstitial cells in experimental models (113).

Other CD4^+^ T cell–associated pathways in CAVD remain less well defined. Regulatory T cells (Tregs) may exert immunoregulatory functions through IL-10 and other suppressive mediators and may dampen excessive myeloid inflammation within the valvular microenvironment. However, Treg accumulation in severe AS does not necessarily indicate effective disease containment, and its functional significance remains to be determined (111).

Collectively, these pathways illustrate that cytokine effects cannot be inferred solely from associations with specific T cell subsets; cellular source, receptor distribution, disease stage, and experimental context may substantially influence their effects on VICs and myeloid cells. Overall, CD4^+^ T cells are associated with inflammatory amplification, myeloid-cell modulation, and immune–stromal crosstalk in CAVD, with the strongest mechanistic evidence involving Th1/IFN-γ and Th17/IL-17A–related pathways (94, 109–111, 114).

### 5.2 CD8^+^ T cells

CD8^+^ T cells are a prominent component of the adaptive immune landscape in CAVD. Substantial CD8^+^ T cell infiltration has been detected in human calcified aortic valves, predominantly localized around calcified nodules, neovessels, and inflammatory cell clusters (20, 31). Single-cell and T cell receptor (TCR) repertoire data have identified clonally expanded effector and memory T cell populations within diseased valves, consistent with local antigen exposure or persistent inflammatory stimulation (20, 91, 115). This spatial and clonal organization supports involvement in sustained local inflammation but does not demonstrate that CD8^+^ T cells initiate calcification (31, 91). Tissue-resident memory-like CD8^+^ T cells may contribute to the persistence of local immune activation, although their functional contribution to calcification remains uncertain (20, 115).

Activated CD8^+^ T cells may influence the CAVD microenvironment through inflammatory and cytotoxic mediators, including IFN-γ, TNF-α, perforin, and granzyme B (20, 31). At the signaling level, IFN-γ engages IFNGR1/2 to activate STAT1 and gamma-activated sequence (GAS)–dependent transcription, whereas TNF-α signals through TNFR–RIPK1/TRADD/TRAF2 to activate NF-κB, thereby promoting TNF and IL6 expression and reinforcing inflammatory transcriptional programs within the valvular microenvironment (20, 31). Perforin and granzyme B may additionally contribute to local cellular injury, danger-associated molecular pattern (DAMP) release, immune-cell recruitment, and ECM remodeling (20, 91).

Overall, current evidence supports CD8^+^ T cell infiltration, activation, tissue residency, and clonal expansion in CAVD but does not establish CD8^+^ T cells as disease initiators. Further studies are needed to determine whether specific CD8^+^ T cell states or effector pathways represent therapeutically actionable targets in CAVD.

### 5.3 B cells

B cells are rarely detected in healthy aortic valves but accumulate selectively in calcified lesions, particularly on the aortic leaflet surface and around neovessels (107). CD20^+^ B cell density positively correlates with transvalvular pressure gradients and calcification burden, supporting an association between local B cell accumulation and disease severity (107).

The potential contribution of B cells to CAVD appears to involve substantial crosstalk with innate immune cells. In human calcified valves, macrophage infiltration correlates with B-cell activating factor receptor (BAFF-R)-expressing B cells, supporting a putative macrophage-BAFF^+^ B cell axis (107). Activated B cells may also function as antigen-presenting cells and participate in reciprocal B-T interactions, potentially reinforcing chronic inflammatory responses (24, 107). Importantly, most current CAVD-specific evidence for B cells remains observational, and the relative contributions of B cell activation, antigen presentation, antibody production, and interactions with macrophages and T cells remain unresolved. Mechanistic studies are therefore required to determine whether specific B cell subsets or B cell–myeloid/T cell interactions directly contribute to fibrocalcific remodeling in CAVD (21, 107) (**Figure 3**).

**Figure 3:**
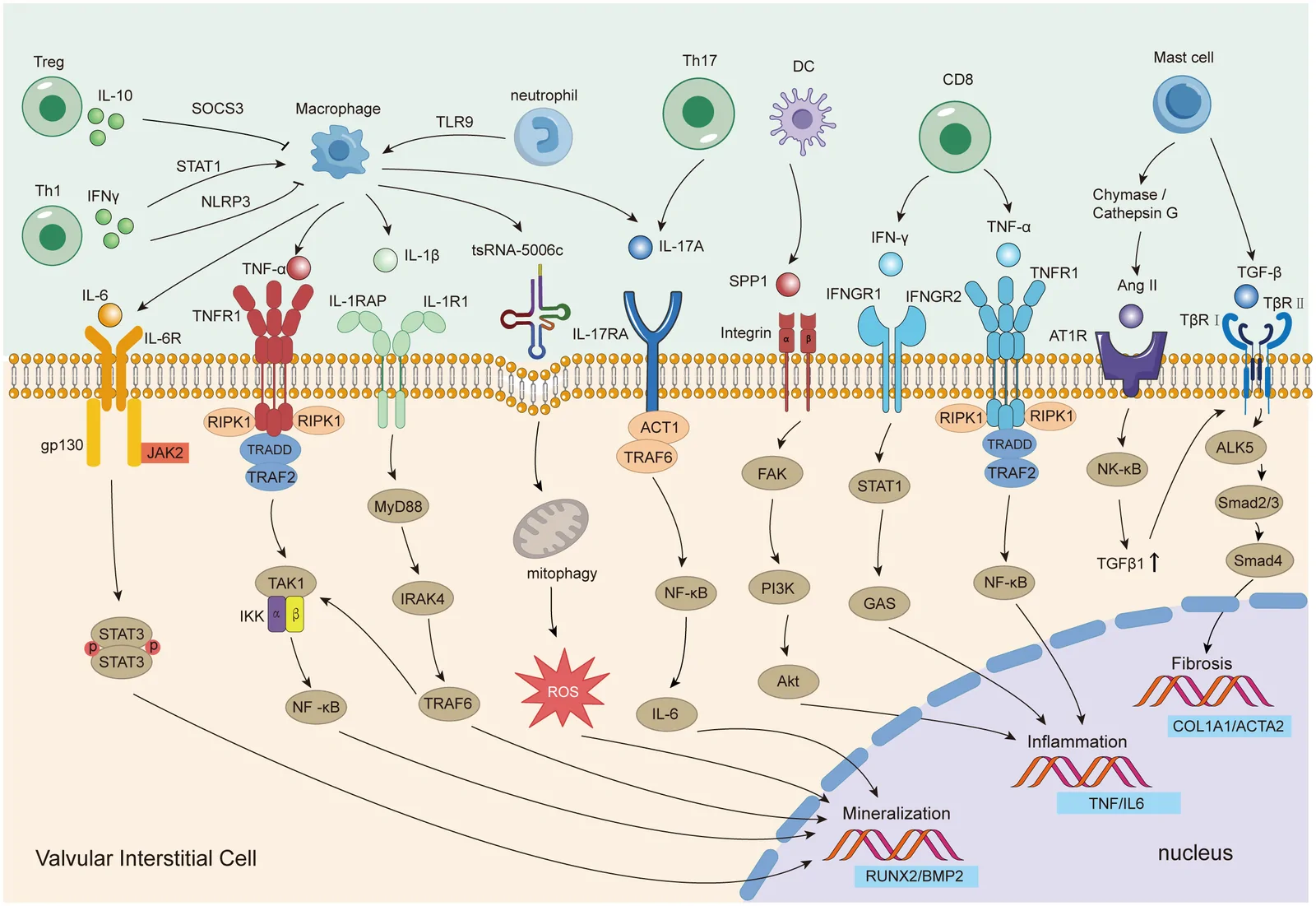
Immune cell–derived signaling network regulating valvular interstitial cell activation in CAVD. Innate and adaptive immune cells release cytokines, proteases, DAMPs, ROS, and NET-associated mediators that converge on VIC-expressed receptors and intracellular signaling cascades. Regulatory T cells (Tregs) and Th1 cells modulate macrophage programming through IL-10–SOCS3 and IFN-γ–STAT1/NLRP3 signaling, respectively, while neutrophil-derived NET-associated products activate macrophages through TLR9. Macrophage-derived IL-6 signals through IL-6R–gp130–JAK2 to activate STAT3; TNF-α engages TNFR1/TNFR2 to trigger the RIPK1–TRADD–TRAF2–TAK1/IKK–NF-κB axis; and IL-1β engages IL-1R1/IL-1RAP to activate the MyD88–IRAK4–TRAF6–NF-κB pathway. Macrophage-derived tsRNA-5006c delivered by extracellular vesicles induces mitophagy and ROS generation. Th17-derived IL-17A engages IL-17RA–ACT1–TRAF6–NF-κB signaling to induce IL-6, whereas DC-derived SPP1 engages integrin–FAK–PI3K/Akt signaling. CD8^+^ T cell–derived IFN-γ and TNF-α signal through IFNGR1/2–STAT1/GAS and TNFR1–RIPK1/TRADD/TRAF2–NF-κB, respectively. Mast cell–derived chymase/cathepsin G generates Ang II, engaging AT1R–NF-κB signaling, while mast cell–derived TGF-β engages TβR–ALK5–Smad2/3/Smad4 signaling and amplifies TGF-β1 expression. These convergent pathways promote transcriptional programs of inflammation (TNF, IL6), fibrosis (COL1A1, ACTA2), and mineralization (RUNX2, BMP2), thereby driving inflammatory, fibrotic, and osteogenic remodeling in the valvular microenvironment. ACT1, NF-κB activator 1; Ang II, angiotensin II; AT1R, angiotensin II receptor type 1; CAVD, calcific aortic valve disease; DAMPs, damage-associated molecular patterns; DC, dendritic cell; GAS, gamma-activated sequence; gp130, glycoprotein 130; IKK, IκB kinase; IL, interleukin; IRAK4, interleukin-1 receptor-associated kinase 4; JAK2, Janus kinase 2; MyD88, myeloid differentiation primary response 88; NET, neutrophil extracellular trap; NF-κB, nuclear factor κB; NLRP3, NOD-like receptor family pyrin domain-containing 3; PI3K, phosphoinositide 3-kinase; RIPK1, receptor-interacting protein kinase 1; ROS, reactive oxygen species; SPP1, secreted phosphoprotein 1; SOCS3, suppressor of cytokine signaling 3; STAT, signal transducer and activator of transcription; TAK1, transforming growth factor-β-activated kinase 1; TβR, transforming growth factor-β receptor; TGF-β, transforming growth factor-β; Th, T helper; TNF, tumor necrosis factor; TNFR, tumor necrosis factor receptor; TRADD, TNFR-associated death domain; TRAF, TNFR-associated factor; Treg, regulatory T cell; tsRNA, tRNA-derived small RNA; VIC, valvular interstitial cell.

## 6 Therapeutic strategies for modifying CAVD progression

The identification of immune–stromal interactions in CAVD has expanded interest in therapeutic strategies beyond conventional lipid-lowering and mineral-targeted approaches (28–30, 116, 117). However, mechanistic involvement does not necessarily establish therapeutic validity. Candidate interventions differ substantially in the strength of CAVD-specific evidence, experimental context, and degree of clinical translation. Accordingly, therapeutic strategies should be considered according to whether their effects have been evaluated in clinical studies, validated in experimental models, or remain primarily mechanistic. This section summarizes conventional and emerging approaches targeting inflammatory, immune, fibrotic, and osteogenic mechanisms, with emphasis on the current level of therapeutic evidence and the limitations that remain before clinical application.

### 6.1 Traditional strategies

Statin therapy was one of the earliest candidate disease-modifying strategies investigated for CAVD. SALTIRE evaluated atorvastatin (28), SEAS evaluated simvastatin plus ezetimibe (29), and ASTRONOMER evaluated rosuvastatin (30). Across these trials, statin therapy did not demonstrate a clinically meaningful reduction in the progression of AS or valvular calcification. Statins also exert lipid-independent immunomodulatory effects, including reduced Th1-cell differentiation and enhanced Th2 development (118, 119), yet these pleiotropic actions did not translate into altered progression of established valvular disease in the trials above. Bone and mineral metabolism trials have also been neutral: the Bisphosphonates and Receptor Activator of Nuclear Factor-κB Ligand Inhibition in Aortic Stenosis trial (SALTIRE II) found that denosumab or alendronic acid did not reduce ^18^F-sodium fluoride uptake or slow hemodynamic deterioration (116, 120), while the Aortic Valve DECalcification (AVADEC) trial did not confirm clinical benefit from combined vitamin K2 and vitamin D supplementation (117). Direct inhibition of hydroxyapatite crystallization represents a further mineral-targeted approach: in a randomized phase 2b trial of patients on hemodialysis, the calcification inhibitor SNF472 slowed the progression of cardiovascular calcification, although valve-specific effects were not separately reported (121).

These neutral clinical outcomes illustrate the difficulty of modifying established CAVD with lipid-lowering or mineral-targeted therapies alone. They also highlight the need to evaluate disease-modifying strategies according to their biological target and stage of disease (28–30, 116, 117). Registered and completed pharmacological trials relevant to CAVD are summarized in **Table 1**, whereas **Table 2** summarizes the proposed functions and evidence levels of major immune-cell populations.

**Table 1:** Registered clinical trials and evidence stratification for immune- or inflammation-targeted therapeutic strategies in CAVD.

Trial ID	Acronym	Phase	Year	Status	Target/pathway	Intervention	Primary result	Evidence tier	Next step
Not registered	SALTIRE	Phase 3	2005 (NEJM)	Completed	Lipid lowering; LDL-C	Atorvastatin 80 mg	Neutral; no halting of AVC or AS progression (n = 155)	D	Closed
NCT00092677	SEAS	Phase 3	2008 (NEJM)	Completed	Lipid lowering; LDL-C	Simvastatin + ezetimibe	Primary composite neutral (HR 0.96); ischemic CV events reduced (HR 0.78, 95% CI 0.63–0.97, P = 0.02); no reduction in aortic-valve events (n = 1, 873)	D	Closed
NCT00800800	ASTRONOMER	Phase 3	2010 (Circulation)	Completed	Lipid lowering; LDL-C	Rosuvastatin 40 mg	Neutral; no reduction in AS progression (n = 269; 134 rosuvastatin vs 135 placebo)	D	Closed
NCT01327846	CANTOS	Phase 3	2017 (NEJM)	Completed	IL-1β/NLRP3 inflammasome	Canakinumab 150 mg SC q3mo	MACE HR 0.85, P = 0.021 (n = 10, 061 post-MI; CAVD not pre-specified)	B	CAVD-enriched trial warranted
NCT05162742	CHIANTI	Phase 3	2022 (start)	Active, not recruiting	NLRP3/microtubule	Colchicine 0.5 mg OD	Primary endpoint 24-mo ΔAVC; trial ongoing, completion expected 2026-12 (n = 150)	B	Awaits completion and full readout
NCT04870424	Co-STAR	Phase 3	2025 (Nat Commun)	Terminated (stroke signal)	NLRP3/microtubule	Colchicine post-TAVR	Primary composite (30-d AF or pacemaker) positive: 10% vs 25%, P = 0.031; subclinical leaflet thrombosis 27% vs 54%, P = 0.007; terminated early for stroke imbalance 8.3% vs 0, P = 0.022 (n = 120)	B	Positive efficacy offset by stroke safety signal; further evaluation warranted
NCT02109614	EAVaLL	Early Phase 1	2014	Withdrawn	Lp(a)-oxPL lipid-inflammatory axis	Extended-release niacin	Withdrawn before enrollment (n = 0)	D	Closed
NCT02132026	SALTIRE II	Phase 2	2021 (Circulation)	Completed	Bone and mineral metabolism; RANKL	Denosumab; alendronic acid	Neutral; no ΔAVC, ΔVmax, or ^18^F-NaF uptake reduction (n = 152)	D	Closed
NCT03243890	AVADEC	NA	2022 (Circulation)	Completed	MGP γ-carboxylation	MK-7 720 µg/d + vitamin D3 25 µg/d	Neutral; no ΔAVC reduction at 24 mo (n = 365 men)	D	Closed
NCT03305536	DECAV-K2	Phase 2	2018 (start)	Unknown (no update since 2019-08)	MGP γ-carboxylation	Vitamin K2 (MK-7) + vitamin D3	No results published; primary endpoints ΔGradient, ΔAVA, and ΔVmax at 3 y (target n = 150)	D	Status uncertain; presumed inactive
NCT04055883	DIP-CAVD	Phase 2	2024 (JACC)	Completed	DPP-4/IGF-1 axis; VIC osteogenic switching	Evogliptin/DA-1229	Neutral primary endpoint (96-wk ΔAVCV); favorable secondary ^18^F-NaF and biomarker signals (n = 228)	B	EVOID-AS Phase 2/3
NCT04913870	ARBAS	Phase 4	2020 (start)	Recruiting	RAAS; fibrosis, LV remodeling	Angiotensin II receptor blocker	Ongoing; completion expected 2027-02 (target n = 144)	B	Awaits readout
NCT05143177	EVOID-AS	Phase 2/3	2022 (start)	Terminated	DPP-4 inhibition; immuno-metabolism	Evogliptin	Terminated; completion 2026-03 (n = 486)	B	Awaits final analysis
NCT05861648	Folic acid trial	Phase 2	2023 (registration)	Not yet recruiting (no update since 2023-05)	One-carbon metabolism; homocysteine	Folic acid	Not initiated (target n = 100); status uncertain	B	Pending
NCT05875675	Pioglitazone trial	Phase 2	2023 (registration)	Not yet recruiting (no update since 2023-05)	PPAR-γ; anti-inflammatory, immune-metabolic	Pioglitazone	Not initiated (target n = 100); status uncertain	B	Pending
NCT02481258	CAVS (Ataciguat Phase II)	Phase 2	2015 (start)	Completed	Oxidized sGC-cGMP-PKG; NO signaling	Ataciguat/HMR1766	Changes in AVC at 6 mo; supported Phase 2b/3 program (n = 35)	A	Advanced to KATALYST-AV
NCT07001800	KATALYST-AV	Phase 2/3	2025 (start)	Recruiting	Oxidized sGC-cGMP-PKG; AVC, AVA decline	Ataciguat	Part A 24-wk ΔAVC positive (reported AHA 2025); Part B dual-primary (ΔAVA, Δpeak VO2 at Wk 48) ongoing (target n = 1, 276, completion 2030-09)	A	Part B ongoing; Phase 3 readout expected 2030

Evidence tiers: A, positive Phase 2 signal supporting Phase 3 advancement; B, clinical data available but primary endpoint neutral, suspended, terminated, or ongoing, requiring refined trial design, CAVD-enriched evaluation, or full publication; C, preclinical only; D, failed in Phase 3 (or withdrawn) due to mechanism mismatch, late-window enrollment, insufficient valve-tissue exposure, or early discontinuation. “Year” denotes trial start year for ongoing or unpublished trials, registration year for trials not yet initiated, or publication year for completed trials with peer-reviewed primary results.

AF, atrial fibrillation; ARB, angiotensin II receptor blocker; ARBAS, Angiotensin Receptor Blockers in Aortic Stenosis; AS, aortic stenosis; AVA, aortic valve area; AVC, aortic valve calcification (Agatston units); AVCV, aortic valve calcium volume; CANTOS, Canakinumab Anti-inflammatory Thrombosis Outcomes Study; CAVS, Calcific Aortic Valve Stenosis; CAVD, calcific aortic valve disease; CHIANTI, Colchicine and Inflammation in Aortic Stenosis; Co-STAR, Colchicine in Aortic Stenosis Undergoing Transcatheter Aortic Valve Replacement; CT, computed tomography; DECAV-K2, Decalcification of the Aortic Valve by Vitamin K2; DIP-CAVD, clinical study to evaluate the efficacy and safety of DA-1229 in patients with calcific aortic valve disease; DPP-4, dipeptidyl peptidase-4; EAVaLL, Early Aortic Valve Lipoprotein(a) Lowering Trial; EVOID-AS, study of evogliptin in calcific aortic valve disease with mild to moderate aortic stenosis; HR, hazard ratio; IGF-1, insulin-like growth factor-1; KATALYST-AV, Ataciguat for Slowing the Progression of Moderate Calcific Aortic Valve Stenosis; LDL-C, low-density lipoprotein cholesterol; Lp(a), lipoprotein(a); MACE, major adverse cardiovascular events; MGP, matrix Gla protein; MK-7, menaquinone-7; NCT, ClinicalTrials.gov identifier; NLRP3, NOD-like receptor family pyrin domain containing 3; NO, nitric oxide; oxPL, oxidized phospholipid; PPAR-γ, peroxisome proliferator-activated receptor gamma; PKG, protein kinase G; RAAS, renin–angiotensin–aldosterone system; RANKL, receptor activator of nuclear factor-κB ligand; SALTIRE, Scottish Aortic Stenosis and Lipid Lowering Trial: Impact on Regression; SALTIRE II, Bisphosphonates and RANKL Inhibition in Aortic Stenosis; SEAS, Simvastatin and Ezetimibe in Aortic Stenosis; sGC, soluble guanylate cyclase; TAVR, transcatheter aortic valve replacement; VIC, valvular interstitial cell; ΔAVA, change in aortic valve area; ΔAVC, change in aortic valve calcification; ΔAVCV, change in aortic valve calcium volume; ΔVmax, change in peak aortic jet velocity.

**Table 2:** Functional positioning and evidence strength of major immune cells in CAVD.

Cell type	Role in CAVD	Key effector molecules	Evidence type	References
Monocytes	Inflammatory initiators and macrophage precursors	TNF-α, IL-6, CCR2/CCL2	Clinical observation, peripheral-blood analysis, and *in vitro* studies	(73, 85)
M1-like macrophages	Amplifiers of inflammation and procalcific VIC signaling	TNF-α, IL-1β, IL-6, NLRP3, EV cargo	Human valve tissue, coculture, EV/small-RNA studies, and animal models	(18, 72, 80, 81)
M2-like macrophages	Repair-associated but potentially profibrotic regulators	IL-10, TGF-β1, Smad2/3	*In vitro*, animal, and human correlative evidence	(17, 21, 22, 88)
DCs	Antigen-presentation bridge between innate and adaptive immunity	MHC-II, CD80/CD86, CD40, IL-12	Human tissue observation and immune-mechanistic inference	(23, 24, 75)
Neutrophils	NET- and elastase-mediated inflammatory amplifiers	NETs, TLR9, neutrophil elastase, MPO, ROS	Clinical observation, valve tissue, *in vitro*, and mouse-model evidence	(78, 100–102)
NK cells	Auxiliary regulators of inflammatory microenvironments	IFN-γ, TNF-related signals, cytotoxic molecules	Transcriptomic infiltration analyses and limited tissue evidence	(21, 33, 38, 49, 79)
Mast cells	Protease-mediated matrix-remodeling cells	Chymase, cathepsin G, tryptase, VEGF	Human histology, clinical association, and mechanistic inference	(19, 76, 77)
Th1 cells	Adaptive inflammatory amplifiers	IFN-γ, TNF-α, T-bet, STAT1	Human tissue, animal, and *in vitro* evidence	(109, 154)
Th17 cells	IL-17A-driven inflammatory and fibrotic amplifiers	IL-17A, IL-17RA, RORγt, CXCL2, CXCL8	Human valve transcriptomics, human-cell experiments, and ApoE-/- mice	(110)
γδ T cells	Rapid inflammatory responders and potential early IL-17A source	IL-17A, IL-23 axis, γδ TCR	Mouse experiments and human valve transcriptomic signals	(114)
Tregs	Immunosuppressive and compensatory regulatory cells	*FOXP3*, IL-10, TGF-β	Mainly clinical and peripheral-blood flow-cytometry evidence	(111)
CD8^+^ T cells	Cytotoxic injury and chronic local immune maintenance	IFN-γ, TNF-α, GZMB, PRF1, TCR expansion	Human valve tissue, single-cell/TCR sequencing, and clinical samples	(20, 31, 91)
B cells	Local humoral and antigen-presenting participants	CD20, BAFF/BAFF-R, immunoglobulins	Human valve histology, immunostaining, and clinical correlation	(107)

ApoE, apolipoprotein E; BAFF, B-cell activating factor; BAFF-R, B-cell activating factor receptor; CAVD, calcific aortic valve disease; CCL2, C-C motif chemokine ligand 2; CCR2, C-C motif chemokine receptor 2; CD8+, cluster of differentiation 8-positive; CD20/40/80/86, cluster of differentiation 20/40/80/86; CXCL2/8, C-X-C motif chemokine ligand 2/8; DCs, dendritic cells; ECM, extracellular matrix; EV, extracellular vesicle; FOXP3, forkhead box P3; GZMB, granzyme B; IFN-γ, interferon-gamma; IL-1β, interleukin-1 beta; IL-6, interleukin-6; IL-10, interleukin-10; IL-12, interleukin-12; IL-17A, interleukin-17A; IL-17RA, interleukin-17 receptor A; IL-23, interleukin-23; M1, classically activated macrophage phenotype; M2, alternatively activated macrophage phenotype; MHC-II, major histocompatibility complex class II; MPO, myeloperoxidase; NETs, neutrophil extracellular traps; NK, natural killer; NLRP3, NOD-like receptor family pyrin domain containing 3; PRF1, perforin 1; RNA, ribonucleic acid; RORγt, retinoic acid receptor-related orphan receptor gamma t; ROS, reactive oxygen species; RUNX2, runt-related transcription factor 2; Smad2/3, SMAD family members 2 and 3; STAT1/3, signal transducer and activator of transcription 1/3; T-bet, T-box transcription factor; TCR, T cell receptor; TGF-β, transforming growth factor-beta; TGF-β1, transforming growth factor-beta 1; Th1/17, T helper 1/17; TLR9, Toll-like receptor 9; TNF-α, tumor necrosis factor-alpha; Tregs, regulatory T cells; VEGF, vascular endothelial growth factor; VIC, valvular interstitial cell; γδ T cells, gamma delta T cells.

### 6.2 Anti-inflammatory strategies

Inflammatory signaling is particularly prominent in early and intermediate CAVD and represents one potential therapeutic entry point. Several experimental approaches have been proposed to modulate inflammatory signaling in CAVD, including cGAS–STING inhibition (122), IL-37/IL-38-related pathways (123, 124), pro-resolving omega-3/resolvin pathways (125, 126), and antioxidant strategies targeting stress-induced inflammatory responses (127). However, the evidence supporting these approaches remains predominantly preclinical, and their therapeutic effects have been demonstrated mainly in experimental models rather than in adequately powered clinical trials. These findings therefore support biological plausibility but should not yet be interpreted as evidence of clinical efficacy. Clinical evidence from other cardiovascular diseases provides useful but indirect support for targeting inflammatory pathways. The Canakinumab Anti-inflammatory Thrombosis Outcomes Study (CANTOS) demonstrated that IL-1β inhibition can reduce recurrent cardiovascular events in patients with established atherosclerotic disease, but it was not designed to evaluate CAVD and therefore cannot establish efficacy against valvular calcification or AS (128). Similarly, the ongoing Colchicine and Inflammation in Aortic Stenosis (CHIANTI) trial provides a more disease-specific test of anti-inflammatory therapy in CAVD, using 18F-sodium fluoride imaging and hemodynamic outcomes (129, 130). Results from such trials will be important for determining whether systemic inflammatory modulation can alter the trajectory of human CAVD rather than simply modify systemic inflammatory biomarkers.

Because established mineralization and structural remodeling may be less responsive to inflammatory modulation, the therapeutic effects of anti-inflammatory interventions may depend on the biological stage of CAVD. In addition, systemic immune modulation may produce adverse effects when inflammatory pathways also participate in host defense or tissue repair. These considerations highlight the need for disease-specific evidence and careful assessment of therapeutic safety before anti-inflammatory strategies can be translated into routine CAVD treatment (4, 130).

### 6.3 Adaptive immune modulation

Adaptive immune dysregulation has been implicated in inflammatory-osteogenic remodeling in CAVD, particularly through Th17-associated cytokine signaling and an impaired regulatory response (110, 111). These findings raise the possibility that pathway-selective immune modulation, including modulation of JAK/STAT signaling or Th17-associated pathways, could be explored as an alternative to broad systemic immunosuppression (131). However, the therapeutic relevance of individual T-cell subsets remains uncertain because cytokine effects vary according to the responding cell type and local tissue context. The context-dependent effects of IFN-γ discussed above further illustrate this complexity. Evidence for B-cell-directed intervention is even less mature, with current CAVD data remaining largely observational. Thus, adaptive immune pathways represent mechanistically supported therapeutic candidates, but their disease-modifying potential remains to be established.

### 6.4 Antifibrotic strategies

Fibrosis and aberrant ECM remodeling are important components of CAVD and may influence VIC phenotype, tissue stiffness, and subsequent mineralization, although fibrosis and calcification are not necessarily temporally or mechanistically coupled. Increased matrix stiffness may further modify VIC mechanotransduction through YAP-dependent and related pathways (62, 63). Lysyl oxidase (LOX)-dependent collagen crosslinking and matrix metalloproteinase activity have therefore been investigated as potential antifibrotic targets, with experimental evidence supporting effects on matrix remodeling and calcification-related phenotypes (64, 65, 132).

However, antifibrotic efficacy should not be equated with reversal of established mineralization. Experimental reduction of myofibroblastic activation or matrix contraction may coexist with persistent calcific deposits, particularly when osteogenic programs and mineral nucleation have become established. Whether modulation of ECM remodeling can translate into slower calcification or improved valve hemodynamics therefore remains to be determined.

### 6.5 Targeting osteogenic conversion

VIC osteogenic reprogramming is an important component of valvular mineralization in CAVD and involves interconnected pathways including RUNX2, BMP2, Wnt/β-catenin, DPP-4, oxidative stress, and metabolic signaling (40, 41, 46, 133, 134). DPP-4 inhibition has received particular interest because evogliptin reduced inflammatory, fibrotic, and calcification-related phenotypes in preclinical models (135); however, clinical testing has not demonstrated a significant reduction in CT-defined aortic valve calcification volume, despite suggestive changes in ^18^F-sodium fluoride positron emission tomography (PET) signals (130, 136). This discordance illustrates the difficulty of translating changes in molecular or imaging activity into clinically meaningful modification of CAVD progression. Other experimental approaches targeting BMP signaling, protein tyrosine phosphatase 1B (PTP1B), autophagy, peroxisome proliferator-activated receptor gamma (PPARγ), and hypoxia-inducible factor 1-alpha (HIF1A) have similarly provided evidence that osteogenic signaling is experimentally modifiable (137–141). Most of these approaches, however, remain at the preclinical stage, and their effects on clinically meaningful CAVD outcomes remain unknown.

### 6.6 Evidence synthesis and translational feasibility of immune-targeted therapies

Taken together, therapeutic evidence in CAVD remains heterogeneous across biological targets and stages of translation. Conventional lipid-lowering and mineral-targeted interventions have undergone clinical evaluation without demonstrating consistent disease-modifying efficacy in established CAVD (28–30, 116, 117). Anti-inflammatory strategies range from CAVD-specific preclinical targets, such as NLRP3 and cGAS–STING, to pathways currently undergoing clinical investigation, whereas adaptive immune, antifibrotic, and most osteogenic interventions remain predominantly preclinical (122, 123, 129, 132, 142). The available evidence therefore supports biological involvement of multiple inflammatory and fibrocalcific pathways, but only a limited number of strategies have progressed to disease-specific clinical testing. This gap between mechanistic evidence and demonstrated clinical benefit remains a central challenge for immune-informed CAVD therapy.

The candidate strategies outlined above share four interrelated translational bottlenecks. Feasibility of sustained target engagement is uncertain in a slowly progressive disease where efficacy likely requires years of continuous exposure, while systemic toxicity is a recurring constraint across classes—IL-1β blockade and NLRP3 modulation compromise innate host defense, JAK inhibitors carry label warnings for serious infections, thromboembolism, and malignancy, and IL-17A blockade is associated with mucocutaneous candidiasis and inflammatory bowel disease exacerbation. The therapeutic window is tightly stage-dependent; interventions targeting inflammatory or fibrotic nodes may be effective only before microcalcification becomes irreversible, as illustrated by the consistent failure of statin and bone-metabolism trials in established lesions (28–30, 116, 117). Patient selection has similarly relied on hemodynamic rather than biological criteria, diluting effect estimates across SALTIRE, SEAS, ASTRONOMER, SALTIRE II, AVADEC, and DIP-CAVD. CANTOS provides proof-of-concept that IL-1β inhibition can reduce atherosclerotic MACE, but no CAVD-specific trial has met its primary endpoint to date, and the DIP-CAVD program indicates that DPP-4 inhibition does not reproducibly slow valvular calcification. **Table 3** consolidates these considerations across the principal immune-targeted strategies, providing a side-by-side comparison of mechanism, evidence maturity, and remaining translational gaps.

**Table 3:** Preclinical and clinical evidence for immune- and inflammation-directed therapeutic targets in CAVD.

Target	Preclinical evidence	Clinical evidence	Main limitation
IL-1β blockade Canakinumab/anakinra	Inhibits NLRP3–IL-1β inflammation and VIC osteogenic remodeling	**CANTOS**: reduced MACE; **not CAVD-specific**	No CAVD RCT; infection risk
NLRP3 inhibition Colchicine	Suppresses inflammasome activation and valve inflammation/calcification	**CHIANTI**: Phase 3, n=150, ongoing	No efficacy results yet
JAK/STAT inhibition Tofacitinib/baricitinib	Suppresses cytokine-driven inflammation and VIC osteogenesis	**No CAVD RCT**	Broad immunosuppression; safety concerns
IL-17A blockade Secukinumab/ixekizumab	Reduces valve inflammation and osteogenic signaling	**No CAVD RCT**	Translational efficacy unproven
DPP-4 inhibition Evogliptin	Reduces inflammation, fibrosis, and VIC calcification	**DIP-CAVD**: Phase 2, n=228; **primary endpoint negative** **EVOID-AS**: ongoing	Efficacy remains uncertain
sGC activation Ataciguat	Restores NO–sGC–cGMP signaling and suppresses osteogenic remodeling	**Phase 2**: approximately 70% slower AVC progression; borderline significance **KATALYST-AV**: Phase 2b/3 ongoing	Small Phase 2; confirmation needed
Valve-targeted delivery PLGA/liposomes/EVs	Enhances local drug delivery and reduces systemic exposure	**Preclinical only**	Delivery, safety, and translational challenges
Statins Atorvastatin/rosuvastatin	Modulates lipid-driven inflammation	**SALTIRE/SEAS/ASTRONOMER**: no convincing slowing of AS progression	No established CAVD disease-modifying effect

Bold type indicates the trial acronym or the principal reported outcome. Clinical evidence is restricted to registered trials in CAVD; entries marked “No CAVD RCT” or “Preclinical only” indicate that no registered randomized trial has evaluated that target. Trial acronyms are defined in the **Table 1** footnote; CANTOS enrolled an atherosclerotic, not a CAVD-enriched, population. Abbreviations: AVC, aortic valve calcification; cGMP, cyclic guanosine monophosphate; DPP-4, dipeptidyl peptidase-4; EVs, extracellular vesicles; IL, interleukin; JAK/STAT, Janus kinase/signal transducer and activator of transcription; MACE, major adverse cardiovascular events; NLRP3, NOD-like receptor family pyrin domain containing 3; NO, nitric oxide; PLGA, poly(lactic-co-glycolic acid); RCT, randomized controlled trial; sGC, soluble guanylate cyclase; VIC, valvular interstitial cell.

## 7 Future perspectives and translational challenges

The therapeutic landscape summarized above highlights a persistent gap between mechanistic evidence and clinically demonstrated disease modification in CAVD. Addressing this gap will require more than the identification of additional inflammatory or osteogenic targets. Future studies should determine which biological features distinguish potentially modifiable disease, when intervention is most likely to be effective, how treatment response can be monitored, and how findings from experimental models can be translated to heterogeneous patient populations. These challenges are closely interconnected and require integration of molecular profiling, imaging, longitudinal phenotyping, and clinically meaningful outcomes.

### 7.1 Multidimensional patient stratification

Disease heterogeneity may contribute to variable therapeutic responses and represents an important consideration for future disease-modifying trials. Clinically similar degrees of aortic stenosis may reflect different combinations of lipid accumulation, inflammatory activity, fibrosis, osteogenic remodeling, and established mineralization. Therapeutic responsiveness may therefore depend not only on disease severity but also on the dominant pathological program within individual valves (4, 116, 117).

Several approaches may help characterize these biological states. 18F-sodium fluoride PET/CT can identify metabolically active microcalcification and may provide information beyond conventional measures of established calcific burden (130, 143). Circulating lipoprotein(a) and oxidized phospholipid levels, inflammatory biomarkers, and circulating immune-cell phenotypes may provide complementary information regarding systemic or valvular disease activity (51, 53, 54, 85, 144). Valve-focused single-cell and spatial transcriptomic approaches may further resolve cellular states and immune–stromal interactions that are not captured by circulating measurements or conventional imaging (32, 33, 38, 49, 106). Genetic association and Mendelian randomization studies implicating pathways involving LPA (52–54, 145, 146) and IL6, ALPL, and NAV1 (147) provide additional evidence that lipid-related, inflammatory, and mineralization-associated mechanisms may contribute to inter-individual differences in CAVD susceptibility and progression.

However, none of these approaches should currently be regarded as validated tools for selecting patients for immune-targeted or other disease-modifying therapies. An important translational question is whether combinations of imaging, circulating biomarkers, genetic information, and tissue-derived molecular signatures can distinguish patients with active and potentially modifiable disease from those with predominantly established fibrocalcific remodeling. Prospective studies with longitudinal follow-up and treatment response assessment will be required to determine the predictive value of such multidimensional profiles (144).

### 7.2 Sex-specific heterogeneity

Sex represents an underappreciated axis of biological heterogeneity in CAVD. Histological, CT, and ^18^F-sodium fluoride PET studies have consistently demonstrated that women exhibit greater fibrotic leaflet remodeling and lower valvular calcification burden, whereas men display denser calcification with higher osteogenic marker expression (RUNX2, BMP2) and more active mineral deposition, at matched hemodynamic severity (148–150). This divergence persists across bicuspid and tricuspid valve phenotypes and is accompanied by sex-specific left ventricular remodeling patterns (148, 151, 152).

At the molecular level, transcriptomic and single-cell studies have identified enrichment of TGF-β-driven extracellular matrix programs in female valves, whereas male valves show preferential activation of osteogenic and innate immune signaling, including TLR and NLRP3 inflammasome components, with higher pro-inflammatory macrophage density adjacent to calcific foci (151, 153). Sex-dependent differences in valvular interstitial cell inflammatory responses to TLR and IFN-γ stimulation have also been reported *in vitro* (154). These differences may be partly mediated by estrogen-mediated suppression and androgen-mediated amplification of the BMP2/RUNX2 osteogenic program, with early menopause and elevated lipoprotein(a) further amplifying AVC risk in women (148, 151, 155, 156). Incorporation of sex as a pre-specified stratification factor is therefore warranted in future disease-modifying trials (151).

### 7.3 Local targeted delivery

The biological heterogeneity of CAVD also raises the possibility that therapeutic intervention should be adapted to disease stage and tissue context. Systemic anti-inflammatory, antifibrotic, or metabolic therapies may be limited by systemic exposure, particularly when their molecular targets also participate in host defense or tissue repair. Valve-targeted nanodelivery systems have therefore been investigated as a potential approach to exploit diseased endothelium or remodeled ECM (157, 158). Extracellular vesicle-based strategies may provide additional opportunities for localized delivery and may also have biomarker applications, although most such strategies remain preclinical (58).

Importantly, local delivery alone is unlikely to overcome the biological heterogeneity of CAVD. The therapeutic payload, target cell, disease stage, and duration of exposure will all need to be considered when evaluating targeted delivery systems. Demonstration of increased valve accumulation should therefore be distinguished from evidence of improved valve function or slowed disease progression.

### 7.4 Biomarkers and dynamic monitoring

Circulating biomarkers may provide minimally invasive tools for disease surveillance and therapeutic response evaluation. Lipoprotein(a) and oxidized phospholipid products are associated with CAVD incidence and progression (51–54, 159), while inflammatory mediators, circulating monocyte phenotypes, and extracellular vesicle cargo may provide indirect information regarding systemic or valvular inflammatory activity (58, 85, 144). Integrating these circulating measurements with multimodal imaging may improve longitudinal characterization of disease activity and treatment response (49, 51, 79, 85, 130).

Nevertheless, most candidate biomarkers remain insufficiently validated as predictors of treatment response or hard clinical outcomes. A major challenge is to determine whether changes in circulating biomarkers or imaging activity precede and predict meaningful changes in valve hemodynamics, rather than merely reflect biological activity. Prospective studies incorporating serial imaging, biomarker measurements, and clinical outcomes will therefore be required before these measures can serve as reliable surrogate endpoints in disease-modifying trials.

### 7.5 Experimental models and human translation

The limitations of experimental models represent another major barrier to translation. ApoE^−/−^ and LDLR^−/−^ models reproduce important aspects of lipid-driven vascular inflammation and can provide mechanistic insights into immune–vascular interactions (160–162), but their susceptibility to systemic dyslipidemia and extra-valvular vascular disease may not fully reproduce the natural history of human CAVD (160). Differences in lipid metabolism, lesion distribution, immune-cell composition, and the timing of calcific remodeling should therefore be considered when extrapolating findings from these models to human aortic valve disease (160, 161).

*In vitro* VIC or VEC systems allow precise manipulation of individual pathways but cannot fully reproduce the multicellular, spatially organized, and mechanically complex valvular microenvironment (47, 71). Human tissue studies provide complementary evidence but have their own limitations. Most available human valve specimens are obtained at advanced stages or at the time of valve replacement, making it difficult to determine whether an observed immune phenotype represents an initiating mechanism, a sustained driver, or a response to established tissue injury and calcification. Cross-sectional single-cell and spatial datasets further provide cellular resolution without necessarily establishing temporal causality. Longitudinal human studies and experimental models that better reproduce disease stage, sex, comorbidity, and immune–stromal organization will therefore be important for validating candidate therapeutic targets (160).

### 7.6 Translational priorities

Current evidence highlights translational priorities for CAVD. First, integrated imaging and biomarker profiling should be used to define disease stage and dominant biological activity before therapeutic intervention, while their ability to predict treatment responses require prospective validation (49, 51, 85, 130, 144). Second, valve-targeted delivery systems should reduce systemic off-target effects while increasing local therapeutic efficacy (58, 157, 158). Third, immune targets such as macrophage recruitment, NLRP3 inflammasome activation, NET formation, and Th17/Treg imbalance should undergo stepwise validation from CAVD-specific mechanistic studies to appropriately designed translational and early clinical studies, rather than being advanced directly on the basis of molecular associations alone. Such targets warrant early-phase trials with mechanism-based endpoints (79, 109–111, 163).

Across these priorities, three methodological principles are particularly important: matching intervention to disease stage, distinguishing target engagement from disease modification, and incorporating clinically relevant outcomes alongside molecular or imaging endpoints. Addressing these issues may help determine which immune pathways represent therapeutically actionable mechanisms rather than merely correlates of established CAVD.

## 8 Conclusion

Accumulating evidence supports an important role for immune-cell infiltration and inflammatory signaling in the fibrocalcific remodeling of CAVD. Rather than acting through a single linear pathway, immune cells interact with VECs, VICs, and the ECM within a dynamic valvular microenvironment. Monocytes and macrophages have the strongest CAVD-specific mechanistic evidence among innate immune populations and can influence inflammatory amplification, matrix remodeling, and procalcific signaling. T-cell subsets, particularly Th1- and Th17-associated pathways, may further shape these processes through cytokine-dependent immune–stromal interactions, whereas the contributions of B cells, DCs, mast cells, neutrophils, and NK cells remain more heterogeneous or less firmly established.

Clinical trials of statin-based and mineral-targeted strategies have not demonstrated consistent disease-modifying efficacy in established CAVD (28–30, 116, 117), underscoring the difficulty of modifying advanced fibrocalcific disease through isolated targeting of systemic lipid or mineral pathways. At the same time, experimental studies of inflammatory, immune, antifibrotic, and osteogenic pathways provide evidence that several components of CAVD biology are potentially modifiable. Whether these effects can be translated into meaningful clinical benefit remains dependent on disease stage, target specificity, patient selection, safety, and the ability to demonstrate changes in valve function rather than molecular or imaging activity alone.

Several limitations continue to constrain interpretation of the current evidence. First, mechanistic findings are derived from heterogeneous sources, including animal models, *in vitro* VIC/VEC systems, single-cell datasets, and predominantly end-stage human valve specimens, each of which captures only part of the disease process. Second, many human studies remain cross-sectional, limiting the ability to determine whether specific immune phenotypes initiate disease progression, contribute to its persistence, or arise secondarily to established tissue injury and calcification. Third, differences between experimental models and human CAVD, together with uncertainty regarding disease stage and patient heterogeneity, complicate translation of promising mechanistic targets into effective therapies (4, 9–11, 21).

Future progress will depend on linking high-dimensional molecular profiling with longitudinal phenotyping and clinically meaningful outcomes. Spatial transcriptomics, CITE-seq, and related approaches may help resolve the cellular organization and functional states of immune–stromal networks, while molecular imaging and circulating biomarkers may enable longitudinal assessment of disease activity (32, 33, 38, 49, 79). However, these technologies will be most informative when used to address specific translational questions: which immune pathways are causally involved, which disease stages remain biologically modifiable, and which patients are most likely to benefit from targeted intervention. Ultimately, a more precise understanding of immune–VEC–VIC interactions may support the development of stage-specific and mechanism-based strategies for delaying CAVD progression, while valve replacement will remain the established treatment for advanced symptomatic AS (58, 79, 88, 157, 158).

## Glossary

ACTA2: Actin alpha 2, smooth muscle
ALPL: Alkaline phosphatase
AS: Aortic stenosis
BAFF: B cell activating factor
BAFF-R: B cell activating factor receptor
BMP2: Bone morphogenetic protein 2
CAVD: Calcific aortic valve disease
cGAS: Cyclic GMP-AMP synthase
CHIP: Clonal hematopoiesis of indeterminate potential
COL1A1: Collagen type I alpha 1 chain
CXCL: C-X-C motif chemokine ligand
DAMPs: Damage-associated molecular patterns
DCs: Dendritic cells
DPP-4: Dipeptidyl peptidase-4
ECM: Extracellular matrix
EndMT: Endothelial-to-mesenchymal transition
EVs: Extracellular vesicles
FOXP3: Forkhead box P3
HIF1A: Hypoxia-inducible factor 1-alpha
ICAM-1: Intercellular adhesion molecule 1
IFN-γ: Interferon-gamma
IGF-1: Insulin-like growth factor-1
JAK: Janus kinase
LOX: Lysyl oxidase
Lp(a): Lipoprotein(a)
MAPK: Mitogen-activated protein kinase
MHC-II: Major histocompatibility complex class II
MMPs: Matrix metalloproteinases
MPO: Myeloperoxidase
NETs: Neutrophil extracellular traps
NF-κB: Nuclear factor kappa B
NK: Natural killer
NLRP3: NOD-like receptor family pyrin domain containing 3
OPN: Osteopontin
oxLDL: Oxidized low-density lipoprotein
oxPL: Oxidized phospholipid
PPAR: Peroxisome proliferator-activated receptor
PRF1: Perforin 1
PTP1B: Protein tyrosine phosphatase 1B
RAAS: Renin-angiotensin-aldosterone system
RORγt: RAR-related orphan receptor gamma t
ROS: Reactive oxygen species
RUNX2: Runt-related transcription factor 2
sGC: Soluble guanylate cyclase
SPP1: Secreted phosphoprotein 1
STAT: Signal transducer and activator of transcription
STING: Stimulator of interferon genes
T-bet: T-box transcription factor
TCR: T cell receptor
TGF-β: Transforming growth factor-beta
TLR: Toll-like receptor
TNF-α: Tumor necrosis factor-alpha
Tregs: Regulatory T cells
VCAM-1: Vascular cell adhesion molecule 1
VECs: Valvular endothelial cells
VEGF: Vascular endothelial growth factor
VICs: Valvular interstitial cells
Wnt: Wingless-related integration site
YAP: Yes-associated protein
